# Perceptions About Technologies That Help Community-Dwelling Older Adults Remain at Home: Qualitative Study

**DOI:** 10.2196/17930

**Published:** 2020-06-04

**Authors:** Henk Verloo, Thomas Kampel, Nicole Vidal, Filipa Pereira

**Affiliations:** 1 School of Health Sciences HES-SO Valais/Wallis Sion Switzerland; 2 University Hospital Lausanne Service of Old Age Psychiatry Prilly Switzerland; 3 La Source, School of Nursing Sciences HES-SO University of Applied Sciences and Arts Western Switzerland Lausanne Switzerland; 4 Conseil Départemental de la Haute Savoie Annecy France

**Keywords:** technology, gerontechnology, photo-elicitation, informal caregivers, cognitive impairment, professional caregivers, interviews, focus groups, content analysis, physical impairment, frailty

## Abstract

**Background:**

The population of Europe is aging rapidly. Most community-dwelling older adults (CDOAs) want to remain in their homes, particularly those experiencing functional decline. Politicians and academics repeatedly praise technological instruments for being the preferred solution for helping older adults with deteriorating health to remain at home.

**Objective:**

This study aimed to understand the perceptions of CDOAs and their informal caregivers (ICs) and professional caregivers (PCs) about technologies that can help keep older adults at home.

**Methods:**

This qualitative study used personal interviews, focus groups, and photo-elicitation interviews to better understand the perceptions of a convenience sample of 68 CDOAs, 21 ICs, and 32 PCs.

**Results:**

A fraction of CDOAs did not perceive technological instruments to be a very useful means of helping them remain at home. However, the ICs and PCs were more positive. The CDOAs preferred and were more willing to adopt technologies related to their mobility and safety and those that would help slow down their cognitive decline. The ICs preferred technological aids that assist in the activities of daily living as well as safety-related technologies for detecting falls and helping to locate disoriented older adults. The PCs preferred integrated communication and information systems to improve collaboration between all stakeholders, housing equipped with technologies to manage complex care, high-performance ancillary equipment to transfer people with reduced mobility, and surveillance systems to ensure safety at home.

**Conclusions:**

Although our study reports that CDOAs have limited interest in innovative technologies to help them remain at home, their technological skills will undoubtedly improve in the future, as will those of ICs and PCs. Technological tools will play an increasingly important role in home health care.

## Introduction

### Background

The population of Europe is aging rapidly [1]. Most countries will have to cope with increasing numbers of frail, community-dwelling older adults (CDOAs) who are losing their autonomy and becoming dependent on assistance [1]. Despite their disabilities, 9 out of 10 CDOAs want to remain at home, even those experiencing significant functional decline and loss of autonomy [2]. In this context, caring for CDOAs has become a major health care, social, economic, and political issue [3]. Demographic transition results in an aging society, with fewer young adults available to support the needs of a dramatically rising number of dependent older adults [4]. The costs of specialist home care and social care to support CDOAs will increase exponentially. Innovation in the management of person-centered care is unavoidable, and it must consider medical history, life expectancy, economic constraints, and society’s expectations for optimal autonomy and mobility [5,6]. Technology can provide proactive solutions for some of the main health and societal problems encountered during aging, such as loss of independence, chronic diseases, psychopathological disorders, and falls at home [6,7]. Despite the omnipresence of technology in modern society, research into its use for improving the daily lives of frail, cognitively impaired CDOAs is limited in naturalistic settings. Technologies can offer innovative ways of improving the health and quality of life of frail CDOAs—or other people losing their autonomy—and help them to remain at home [6].

Technology aimed at older adults is usually termed gerontechnology (which this paper will use as a synonym of technology). It is destined to have an important future role in providing support and solutions, monitoring health status to optimize autonomy, and improving the quality of life of CDOAs, regardless of their level of dependency [8,9]. Gerontechnologies usually have two main, and in most cases, complementary purposes: (1) strengthening the (objective) monitoring of older adults by remotely collecting large amounts of data to alert their informal caregivers (ICs) or professional caregivers (PCs) about health decompensation, so that they can anticipate and implement appropriate intervention strategies, and (2) remotely intervening with CDOAs for the first interaction (using voice or images) [10].

Cornet and Carre [11] mentioned that certain technologies could help maintain levels of autonomy despite debilitating age-related diseases, improve social cohesion, reduce loneliness, compensate for declining capacities, help both ICs and PCs, and, finally, reduce some of the effects of pathological aging. Science and technology will also serve CDOAs by ensuring safer environments, promoting and strengthening their independence and quality of life, and supporting both their ICs and PCs [11-13]. New devices should make it possible to alert ICs and PCs about the risks of health decompensation so that they can anticipate and implement appropriate intervention strategies or even intervene remotely, thus reducing the burden on both groups of caregivers [14]. Several studies have shown that telehealth (ie, technological solutions that enable remote monitoring of health status) is effective in improving the well-being and quality of life of CDOAs [15-17]. Technological instruments also allow users to participate actively in their health care and treatment follow-up [18,19]. Despite this potential, the perceptions of CDOAs and their ICs and PCs about the usefulness of technologies in maintaining their health status and helping them to age well at home have only rarely been examined. Previous studies have demonstrated that gerontechnologies are seldom used actively by older adults themselves, and indeed, the vast majority were unaware of their existence [20,21]. However, in recent years, there has been a resurgence of creativity in this field, involving academic researchers, private companies, and public health care services. Questioning the utility of new technologies, their potential uses, and their acceptance and limitations remains relevant [22].

How are technologies that help them remain at home perceived by CDOAs, their ICs, and PCs? More specifically, how do they rate their actual and potential utility?

### Objectives

This study, overall, aimed to examine and understand the perceptions and utility of these technologies among physically and cognitively dependent CDOAs, their ICs, and PCs.

### Theoretical Framework

This study was guided by the theoretical framework developed by Peek et al [23] and is thus based on the basic components of accepting technology as stated in the results and finding it useful. This framework integrates several factors: (1) utility and ease of use; (2) covering basic needs with technologies; (3) the sense, meaning, acceptance, and value of these technologies; and (4) technologies that help regain or maintain autonomy. According to Peek et al [24], technology can play a role in helping CDOAs remain independent, active, and healthy for as long as possible. Previous studies indicate that the current models of technology acceptance lacked essential predictors, specifically for older adults [25,26].

## Methods

### Design

This international multicenter study used a qualitative design to collect data on the perceptions of CDOAs, their ICs, and PCs using one-to-one interviews, focus groups (FGs), and photo-elicitation interviews (PEIs). Reporting on the study was based on the checklist for the explicit and comprehensive reporting of qualitative studies [27].

### Population and Settings

The study population included CDOA men and women, aged ≥65 years, living in the French department of Haute-Savoie or the French-speaking part of Switzerland’s canton Valais, and with a medical prescription or indication for home care. The reason for examining perspectives in 2 countries was not to compare their populations but rather to explore a common issue together (especially in a border area) and codevelop implementable recommendations for clinical practice and technology companies. ICs and PCs involved in clinical practice and employed in 1 of the 5 different community health care centers (2 in France and 3 in Switzerland) were also questioned.

### Participant Recruitment

A similar sampling strategy was applied to CDOAs in both countries. On the basis of the comprehensive geriatric assessment database in the Autonomy Scale of Gerontology and Iso-Resource Groups (AGGIR) [28], researchers categorized the functional state of potential participants into 1 of the 3 relevant categories: (1) independent CDOA sustained with meals and cleaning services, (2) mainly physically impaired CDOA, or (3) mainly mild-to-moderately cognitively impaired CDOA. CDOAs who were unable to understand and speak French or who lacked the capacity for discernment to consent were excluded.

The AGGIR scale includes 2 types of variables: discriminative and illustrative. Discriminative variables evaluate coherence, orientation, washing, dressing, feeding, excretion, transfers, moving indoors, moving outdoors, and remote communication. These elements are assessed via a clinical evaluation carried out in the home of the CDOA by a nurse, who chooses from among 3 criteria for each variable: (1) the person can complete the activity alone, (2) the person can complete the activity partially, or (3) the person cannot complete the activity. Each criterion is also evaluated based on whether the person carries out the activity spontaneously, totally, usually, and correctly.

The AGGIR scale is the official national tool in France for comprehensive geriatric assessment under home health care settings. It classifies the geriatric profiles of home-dwelling adults into 6 categories (Groupes Iso-ressources; GIRs). GIRs 5 and 6 designate independent CDOAs who only receive minimal home help, such as meals, cleaning, and shopping. GIRs 3 and 4 designate CDOAs with mostly physical impairments, and GIRs 1 and 2 designate CDOAs with mostly cognitive impairments but also some physical impairments. The AGGIR tool uses clinical observations and questions ICs and older adults themselves on their need for assistance in the essential activities of daily living (ADL). Indeed, AGGIR is based on the topics in the Katz ADL scale, which also classifies CDOAs as independent or physically and/or cognitively impaired. Our study did not use the mini-mental state examination. The research team’s geriatrician (NV) clinically judged the participants’ physical and mental health statuses. In agreement with the ethical committee’s expectations concerning the use of existing clinical data, psychiatric diagnoses were not explored prospectively, as this was beyond the aim of this study. A detailed explanation of the tool is provided in Multimedia Appendix 1.

All 5 centers used randomized sampling to select CDOAs in their respective AGGIR categories (independent, physically impaired CDOAs, and cognitively impaired CDOAs). Multimedia Appendix 1 presents detailed information on categorization using the AGGIR tool. In addition, significant ICs, designated by their CDOAs, were asked to participate in a personal interview and a PEI. The number of ICs recruited was proportional to the CDOA dependency classifications. In collaboration with their supervisors at the 5 community health care centers, purposive samples of PCs involved in direct daily care were invited to participate in 1 of 4 FGs.

### Data Collection Procedure

**Figure 1:**
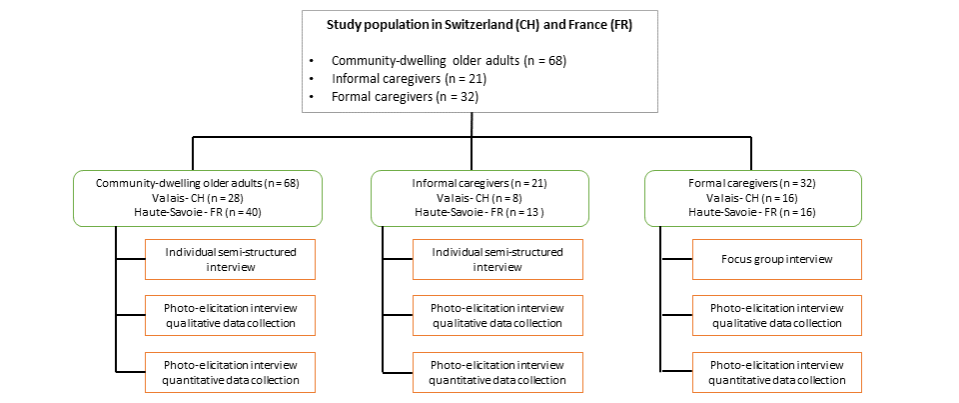
Data collection procedure among the community-dwelling older adults, informal caregivers, and formal caregivers.

This study was approved by the Human Research Ethics Committee of the Canton Vaud (CER-VD – 2017/000789), Switzerland, and the French Data Protection Authority. Data collection took place from September 2017 to June 2018. After the CDOAs, their ICs, and PCs had given their written informed consent to participate, the study nurse collected the participants’ sociodemographic, professional, and health status data (only for the CDOAs) and conducted the scheduled interviews (Figure 1).

#### Community-Dwelling Older Adults

A randomized sample of CDOAs was selected from the 3 categories defined using the AGGIR tool (ie, independent, physically impaired CDOAs, or cognitively impaired CDOAs).

Thus, CDOAs with no limitations according to the AGGIR tool were placed in the independent group, those with physical limitations (eg, traveling and dressing) were categorized in the physically impaired group, and those with cognitive limitations (eg, communication and orientation) joined the cognitively impaired group. Older adults with cognitive and physical limitations were included in the group corresponding to their most pronounced limitation.

Candidates were contacted by telephone and invited to participate. Potential participants received written information about the study before any data were collected. After 48 hours to reflect, a research assistant contacted the potential participants by telephone to address any queries they may have and to obtain their verbal consent to participate in the study. On their acceptance, the assistant made an appointment with the CDOAs for providing more information about the study and to collect the signed consent to participate in the study, before the interview stage. On the day of the interview, the interviewer verified that the participants understood all the information and the implications of participation. Data collection used 10 photographs of relevant technologies and an interview guide developed from a literature review [29]. Audio recordings of the interviews were made.

#### Informal Caregivers

The CDOAs identified their significant ICs. Theoretical samples of 3 to 4 ICs per type of CDOA (independent, physically impaired, or cognitively impaired) were selected by each of the 5 community health care centers, and they were invited to participate in a personal interview and a PEI. Those without the capacity for discernment were excluded. Audio recordings of the interviews were made.

#### Professional Caregivers

In collaboration with the supervisors at the 5 community health care centers, 4 FGs were organized (2 in France and 2 in Switzerland), each with 7 to 8 PCs. The research team proposed meeting dates and times for the FGs. The PCs who were willing to participate signed the consent form beforehand, and audio recordings of the FG discussions were made.

### Data Collection Instruments

The research team developed and tested guides for the one-to-one interviews and PEIs for the CDOAs and ICs and for the FGs. The interview guides encouraged the participants to discuss relevant research issues by asking open-ended questions. The interviewer could reformulate, reorganize, or clarify questions to gain a deeper understanding of the participant’s answers. The guides also helped the participants to better identify practical technologies that might improve health care [30,31]. Data collection using PEIs involved including photographs of relevant technologies into the interview [32,33]. The PEI guide contained 10 photographs, selected using the research team’s empirical expertise (Multimedia Appendix 2), from a recent classification of gerontechnologies [29]. The PEI guide was used at the end of the personal interviews and FG discussions. Each participant was asked to select one or more technologies that they thought would be useful to the 3 separate groups of participants (CDOAs, ICs, and PCs). Each photograph was presented in turn with information on the respective technology. The CDOAs were asked, “What do you think of this technology? What could you use it for?” When all the photographs were laid out on the table, they were asked to choose the technology that seemed the most useful or acceptable in their daily life. The ICs were asked, “What do you think about this technology? How could it be useful to you as an informal caregiver? Among all these photographs, could you choose the one that seems the most useful in your daily life?” The PCs were asked, “What do you think when you see this technology? How could you use it? Which of these technologies would you retain, whether for the help it could provide you or for the help it could provide the clients you work with?” Multimedia Appendix 2 presents the technologies included in the PEI.

### Data Analysis

Descriptive statistics were compiled to describe the participants’ sociodemographic, health, and professional characteristics as well as the technologies selected. Statistical analyses were performed using the IBM Statistical Package for the Social Sciences 25.0 [34]. Personal interviews, PEIs, and FG discussions were transcribed and analyzed using a qualitative content analysis approach [35,36] using NVivo 12 (Qualitative Software International) software [37]. The PEIs were analyzed in parallel using a transversal iterative process and by developing emerging themes. We adopted the approach described by Graneheim and Lundman [35] to ensure the reliability of the results. The interview transcripts were read several times to obtain a sense of the whole. Given the context, units of meaning were condensed into descriptions close to those of the transcripts’ contents and, as far as possible, to an interpretation of their underlying meaning (the latent content). Data were condensed for analysis and examination of their content based on the one-to-one interview guide. Once condensed, the units of meaning were considered as a whole and were abstracted into themes. An 8-hour process of reflection and discussion by the research team resulted in an agreed set of themes. The results were presented in the light of the categories taken from the condensed interviews, and the occurrences selected were illustrated with significant examples, including transcriptions from the interviews. For reasons of confidentiality, reporting considered the CDOAs, ICs, and PCs as 3 groups, without distinguishing health care centers or nationalities.

## Results

### Samples

The study sample was composed of 68 CDOAs, 21 ICs, and 32 PCs. The distribution between the French and Swiss centers was 40 and 28 CDOAs, 13 and 9 ICs, and 16 and 16 PCs, respectively.

### Sociodemographic Data

#### Community-Dwelling Older Adults

Of the 68 CDOAs, almost three-quarters were women (50/68, 74%), with an average age of 82.3 years (SD 7.2; median 82); approximately two-thirds (41/68, 60%) lived in urban areas. Table 1 presents their sociodemographic data and dependency categorization based on the AGGIR scale (Multimedia Appendix 1).

#### Informal Caregivers

The average age of the 21 ICs was 68.4 years (SD 13.8; median 68), with 16 women and 5 men. Most were retired (n=13), lived in urban settings (n=13), and took care of a loved one with a cognitive impairment (n=12) who required partial assistance with the ADL (n=6; Table 2).

#### Formal Professional Caregivers

A total of 4 FGs of 8 participants each brought together 32 PCs, all employed at community health care centers. Their average age was 46.7 years (SD 9.4; median 47) and most were female. Their varied professional roles included physicians, nurses, social workers, nursing assistants, care assistants, and occupational therapists (Table 3).

**Table 1:** The sociodemographic characteristics and functional status of community-dwelling older adults (N=68).

Sociodemographic characteristics and functional status		Values
**Age (years)**		
	Mean (SD)	82.34 (7.2)
	Range	64-95
**Sex, n (%)**		
	Male	18 (26)
	Female	50 (74)
**Place of residence, n (%)**		
	Rural	27 (40)
	Urban	41 (60)
**Level of independence, n (%)**		
	Independent	24 (35)
	Physically impaired	23 (34)
	Cognitively impaired	21 (31)

**Table 2:** The sociodemographic characteristics of informal caregivers (N=21).

Sociodemographic characteristics		Value
**Age (years)**		
	Mean (SD)	68.4 (13.4)
	Range	43-88
**Sex, n (%)**		
	Male	5 (24)
	Female	16 (76)
**Loved one’s level of independence, n (%)**		
	Independent loved one	3 (14)
	Physically impaired loved one	6 (29)
	Cognitively impaired loved one	12 (57)
**Relationship with loved one, n (%)**		
	Spouse	9 (43)
	Daughter/son	9 (43)
	Brother	1 (5)
	Friend	2 (9)
**Occupation, n (%)**		
	Professionally active	8 (38)
	Retired	13 (62)
**Place of residence, n (%)**		
	Rural	8 (38)
	Urban	13 (62)

**Table 3:** The sociodemographic and professional characteristics of professional caregivers (N=32).

Sociodemographic and professional characteristics		Value
**Age (years)**		
	Mean (SD)	46.7 (9.1)
	Range	25-60
**Sex, n (%)**		
	Male	3 (9)
	Female	29 (91)
**Profession, n (%)**		
	Nurses	11 (34)
	Social workers	2 (6)
	Occupational therapists	1 (3)
	Physicians	3 (10)
	Care assistants	10 (31)
	Nursing assistants	3 (10)
	Supervisors	2 (6)

### Findings

We recorded a total of 40 hours and 53 min of personal interviews and PEIs with CDOAs (mean 36 min, SD 13 min; median 35 min; minimum 16 min and maximum 76 min) and 12 hours and 25 min with their ICs (mean 35 min, SD 13 min; median 34 min; minimum 13 and maximum 65 min). We recorded a further 4 hours and 50 min of interviews with the 4 FGs (mean 75 min, SD 6 min; median 72 min; minimum 71 min and maximum 85 min).

In light of the theoretical framework adopted [24], we applied our deductive content analysis along three thematic axes: (1) usefulness and meaning of technology to support the needs of CDOAs, (2) strategies to increase (ease of) technology use among direct and supporting stakeholders, and (3) acceptability of using technology to remain at home among direct and indirect users.

#### Usefulness and Meaning of Technology to Support the Needs of Community-Dwelling Older Adults

##### Community-Dwelling Older Adults

CDOAs perceived technology as something that could be useful, especially for other older adults with health problems, but they rarely saw any sense in using them themselves, except when they had significant difficulties with their ADLs. CDOA 6 said that technology was:

For people who can’t get anything done alone.

CDOA 63 thought it might be needed in future:

Not for the moment, anyway. Maybe in a few years, yes, let’s say, if I have problems in a few years, because I have...well, because of my age!”

CDOA 53 had negative perceptions:

Because all these...these modern techniques, and everything, don’t interest me much. Because it doesn’t match my past life at all.

CDOA 73, however, held an opposing view:

But if something can make life easier, why not? At my age, now’s the time to try.

The concept of technology, in general, was often misunderstood. CDOA 75 exemplified this:

Technologies? What do you mean by technology?

Some CDOAs stated that technology could be useful but not in their current situation. CDOA 9 stated that they did not see any need for it in their current situation:

It works automatically, huh? Of course, but for the moment, it’s not for me.

CDOA 13 thought that some devices might be useful to them if they had more severe health problems:

I think that I’d have to be really bad, huh, to put this thing on.

The (positive or negative) interest in technologies was also often stated by this group. CDOA 9’s positive perceptions of technology were typical:

Well, I think it’s...it’s 2017; I think it’s a necessity, isn’t it?

CDOA 85 spoke out against technology:

And then, it’s no good at all. Sometimes it’s completely harmful, like when things are made available to people, like me, who aren’t always able to understand.

Among CDOAs with mainly cognitive impairments, the word *technology*, without context, was not really understood. As CDOA 82 said:

I don’t know what that means.

The relatively *independent* group mainly spoke about how technology could be useful for others. CDOA 7 gave their opinion:

Well, it’s good for people who don’t have many visitors or people who don’t get any help from their families. And I’m not one of them. So then, no.

Misunderstandings about the term and the meaning of *technology* itself, as in the other groups, came up regularly. CDOA 10 expressed this as a lack of knowledge:

I don’t know which technologies, that’s the problem. That’s because I don’t understand everything...I don’t get it...because, well, I know there are a lot of things now, but I don’t know everything about them.

Positive and negative assumptions appeared in similar proportions among the independent group, as they did for the other 2 groups. For example, CDOA 72 gave their opinion on the internet:

Today, what I don’t agree with is the internet. It’s going to destroy the planet. That’s it, one way or another because it is so dangerous, especially because of those who know it very well, they could flatten the whole world. So, I’m really not interested in the internet; so I’m not interested in computers either.

CDOA 83 shared their positive perception:

If it’s easier for us, if it can help us out, why not, eh?

##### Informal Caregivers

Some ICs considered technologies potentially useful in helping CDOAs remain at home, whereas others considered them as indispensable. Their perceptions on the usefulness of a technology seemed to depend mainly on their current state of health, their own openness to technologies, and the costs involved. Different ICs expressed these aspects as follows:

The thing that might encourage me is if it, err, her case worsened.

IC 38

She has already been offered them, but she doesn’t want them.

IC 56

Yes, change. I think if we change things, I think it would be complicated for her.

IC 38

Oh, well, that must be very expensive.

IC 43

##### Professional Caregivers

PCs perceived technology as useful, but they specified that there was the risk of creating distance in their relationship with the persons in their care and that technology must complete and complement a PC’s work, not replace it:

It can only be complementary. At some point, it makes sense if a real person has to be involved.

FG 2

Moreover, the indication chosen for the use of a specific technology should not only reassure ICs:

What worries me is situations like this, where the children have even put cameras in the bedroom, not to monitor their parents, but rather to reassure themselves.

FG 2

Technologies that could be introduced to enhance the daily lives of CDOAs include furniture with home automation technologies, suitably adapted public transport, and an elderly friendly urban architecture in environments that facilitate transport mobility:

We often have patients who have to take taxis or be driven to hospital or to a medical consultation in town...It’s a real transportation problem.

FG 2

Fifteen years ago, they were all at home, they didn’t move. Now you see some, I don’t know how many, in the city center, using their rollators. It’s pretty impressive. Soon, they’ll invent rollator parking lots.

FG 1

Urban living could also be rethought, perhaps by setting up social centers for CDOAs, on the same principle as youth centers:

Architecturally, it would have to be equipped with ramps for the handicapped, for the rooms where they can meet up. As we do for youth centers, why not build a place for older people with physical difficulties?

FG 1

PCs highlighted their lack of knowledge about the technological devices available on the market and their lack of training on how to pass on skills to CDOAs and ICs:

I don’t have much knowledge about this. It’s true and it makes sense that some training on this wouldn’t be bad.

FG 2

#### Strategies to Increase Ease of Technology Use Among Direct Users and Supporting Stakeholders

##### Community-Dwelling Older Adults

Encouragement from children and grandchildren, financial help for purchasing technology, and increasing feelings of safety were the main strategies identified by CDOAs themselves for improving the ease of use among their peers. For the groups with mainly *physical* and *cognitive impairments*, encouragement to use technology by *children and grandchildren* was mentioned as an important strategy. Family members often gift devices that they no longer use to their older adult relatives. Children choosing devices, such as remote alarms, was also a facilitator of technology use. CDOAs mentioned that receiving financial aid for the acquisition of a device was a facilitator, as was receiving recommendations from PCs. CDOA 35 exemplified the assistance received from PCs:

There is a home for the blind; they proposed it to me. And they sold me the [talking] watch.

CDOA 76 stated:

Yeah, that’s right. So, my granddaughter has a tablet, and my son put my number into it.

Several facilitators to improve beliefs about and attitudes toward technology emerged during the interviews, one being that a device can improve feelings of safety. Financial assistance can be considered a facilitator because it influences people’s willingness to acquire a device. Indeed, for the group of *independent* CDOAs, *financial support* for buying a device and receiving help on usage were the main facilitators of their use. CDOA 84 explained their situation:

Listen, my son gave me this mobile; I didn’t want it. And he said to me, “It’ll be easy.” Anyway, there you go.

CDOA 83 said:

Someone should come and explain and turn it on and say “You have to do this and do that.”

Again, with regard to beliefs and attitudes, a technology that made participants *feel safer* was the main facilitator of use among this group. CDOA 84 explained this in relation to their personal situation:

And then I was unsure, and I was thinking about it, but as I said, I was putting it off and putting it off. And then there’s the personal side, you see? I have great neighbors, but the young ones work, and the old ones are no stronger than me, right? So, I said, “Oh, well!” and then it’s a bit of a safety thing, even though, as I said, I hope I’ll never need it.

*Devices’ properties*, such as ease of use, also influenced CDOAs. CDOA 10 described using a stair-lift:

Yes, usage. You press a button; there’s nothing complicated, eh? There are knobs, one at the top, one at the bottom, so it depends on where you are. Well, you grab the knob. And then, if you go up the stairs, you use the stair-lift device.

##### Informal Caregivers

Some ICs supported the use of technologies if they found them useful and easy to use. As IC 43 said:

If it is a technology that can help me, I will accept it. If it can lighten the burden of helping her.

Surprisingly, other ICs would only introduce certain technologies if the CDOA’s state of health deteriorated. Nevertheless, ICs often felt that they lacked the knowledge to use technologies properly. As IC 71 said:

I like the technological side, but I just have one problem with it. It’s clear that people who weren’t born in the technological age, or who haven’t got themselves up to date by 50 or thereabouts (are going to have trouble). I even find that I’m often overwhelmed.

ICs also noted their relatives’ lack of motivation about the introduction of new technologies:

Ah, well, it’s certainly a very good thing, but she still has to want to do it. She’s not on board with all that’s modern. So, she’ll say no to that

IC 43

The ease of use of technologies was overrided, as perceived by the ICs, by the fear of making mistakes when using them or of infringing on the CDOA’s privacy and autonomy.

##### Professional Caregivers

PCs mentioned that the context of technology implementation was important in its ease of use:

Older adults just need to be interested in new technologies. They are pushed towards it by their children—by their grandchildren, especially.

FG 2

Sometimes a neighbor will say, “You know, I’ve got a rollator, and it goes great,” and that’s it. It’s not some young woman who comes in and says, “This is what you need.”

FG 1

Indeed, technologies were accepted more readily if relatives and ICs were involved:

Sometimes they actually need their family’s support in order to be reassured.

FG 1

Ease of use also seemed important:

There are some things that could improve autonomy as long as they remain simple and easy for people to use.

FG 2

According to PCs, the CDOAs who had either found a technology useful or had used technology in their working lives were more willing to use the proposed technologies:

Some CDOAs are familiar with Skype, for example, using it with grandchildren abroad or even in another city.

FG 3

The elderly population that needs these aids now, is often a population that is unaware of the digital revolution.

FG 4

Although the point of view of individuals and the context in which technologies are implemented tend to be the factors that promote technology use among CDOAs, the cost involved in acquiring different technologies and the different steps required to obtain them can be seen as barriers:

And I find that they’re always scared that “It’s going to be expensive; I can’t.” Sometimes they don’t realize the means they still have, whether financial or not.

FG 4

The emotions associated with technology use can also be a barrier when they generate stress and fear:

The more things or equipment involved, the greater the source of stress, for the patient and the family.

FG 4

You get to a certain point in the aging process where people are not necessarily very accepting of their loss of autonomy. And consequently, in accepting any assistance that might be provided to them.

FG 4

#### Acceptability of Using Technology to Remain at Home Among Direct and Indirect Users

##### Community-Dwelling Older Adults

For all groups of CDOAs, the acceptability of using technologies was influenced by the self-developed strategy for achieving this purpose, difficulties in using the device, and the technology’s cost. As an example of avoiding technology use, several CDOAs said that a simple bedside lamp gave out sufficient light to get to the toilet. The CDOAs found no reason, for example, to replace the home helps who came to clean their home with a robot vacuum cleaner. Help provided directly by humans was the preferred option for many CDOAs. CDOA 13’s thoughts on electronic pill dispensers began with a deep breath:

I’m telling you, I don’t feel the need for one. Oh! If I have something special, my daughter still comes, three times a week, and she is a nurse.

The CDOAs with mainly *physical impairments* did not find some device properties to be user-friendly, and multiple participants considered this to be the main obstacle. CDOA 35 complained about their tablet:

Because handling this device is very complicated for me, you see? I have to turn it all over the place to find out what is going on...No, I’m just not interested.

Another barrier was the cost of acquiring a new technology, as explained by CDOA 6:

Oh, yes, because I can’t say I’m going to buy anything, in addition to what we have to buy every day, because we can’t afford it.

For the CDOA group with mainly *cognitive impairments*, the main barrier was the difficulty in using technologies. This was reflected by CDOA 20:

So, to start with, I don’t want a computer. That’s out of the question; I don’t want a computer. Well because I wouldn’t know how to use it. That’s why.

In several interviews, the group members were found to prefer human contact over technological assistance. CDOA 76 expressed doubts about robots:

Because there’s a person, the nurse, working; a robot isn’t human, whereas a nurse can talk, say something nice, give you a little smile. But a robot’s just ”Crash! Bang!”’

Financial hardship was also consistently mentioned as a barrier to technology use. CDOA 76 said:

Yeah, robots. Yeah, but you need the means to buy one.

For the *independent* CDOA group, financial and usage difficulties were also the most frequently mentioned obstacles. Regarding financial aspects, CDOA 10 said:

Well, let’s say, it’s a good thing. It’s good, but it must have a cost. Well, yes because...I still have to pay for the nursing home; don’t forget that. My husband’s pension, unfortunately, only pays for half of it.

Regarding usage difficulties, CDOA 10 described their tablet:

Right now, I do not know how to use it. My daughter, she’s got one. My grandsons have one each, but so far I’m not interested.

Technologies designed to promote older adults’ home support, such as fall detectors, GPS, and brain training, are the ones that are most frequently accepted by CDOAs. CDOA 73 explained their acceptance:

But we don’t know if I’ll ever be able to...it’s because I’m a little scared, that’s why I don’t go out alone. I’m a little scared to cross the street. So that’s why it’s really the fall detector that would suit me best.

Regarding brain training, CDOA 63 explained:

Well, because by...improving your powers of concentration, developing your vocabulary, anyway because of the propositions that are there, I think it’s good. That’s why.

##### Informal Caregivers

Perceptions about the acceptability of technology use were significantly influenced by safety, ease of use, keeping CDOAs autonomous, and the costs of devices.

ICs explained how the acceptability of technologies was related to the safety of their loved ones. This result can be explained by the fact that of the 21 ICs interviewed, 12 were relatives of CDOAs with mainly cognitive impairments. Thus, the technologies chosen by the ICs were very similar to those chosen by the group of mainly cognitively impaired older adults. Opinions of the ICs regarding the acceptability of the technologies presented during the PEI interviews are as follows. For the light path selected by several ICs, the caregivers said:

Yes, I think that is really good. Well because my mum gets up at night, she can’t find the light.

IC 42

That’s right, because I see he gets lost going to the toilet.

IC 44

Concerning the fall detector, accepted by about one-third of the ICs, they said:

Yes, that could be the most useful there. [...] in the future there could be others [devices].

IC 43

Maybe rather the fall detector, I’d say.

IC 55

The ICs were more reluctant about the electronic pill dispenser:

Ah, well [...], it could be useful because I want him to have a pillbox; the nurses come to put the pills in. But if he doesn’t think about it—if I don’t think about it, he doesn’t think about it—then it’s not much use.

IC 29

Yeah, it’s not bad, but I think it’s pretty expensive for how much we use it. As long as we’re here, well, they’re taken at mealtimes, so I’m here [laughs].

IC 40

[…] In my mother’s case [...] she’d be unable to take her medication alone.

IC 42

The ICs had diverse opinions about robot vacuum cleaners:

For me, these robots are good. You shouldn’t have anything on the ground [laughs] [...] You shouldn’t; it doesn’t go in corners, it’s round [laughs].

IC 29

Well, it depends on how much it costs, huh?

IC 38

Not essential, in my opinion.

IC 40

[….] I think not. No, it might be more dangerous than useful.

IC 42

As for the opinions on the service robot, the ICs said:

Yes, listen, yes, it’s good, but in the end, a human being is still better.

IC 23

[…] I find it rather stressful.

IC 29

Opinions on the usefulness of a GPS bracelet could be summed up by this statement:

That, I, I’m for that. If she ever decided to go somewhere, it would let her know where she was...it would let us know where she was.

IC 38

Only a fraction of the ICs found the touchpad to be an interesting technology. There were different opinions on this:

No, so my mom, my mom... she’s not interested.

IC 23

It might make things easier for him, but since he was not very enthusiastic, he quickly lost patience.

IC 29

Uh, no [laughs], there. Uh, I think it’s impossible. Because even a mobile phone would be complicated to use, so...He still manages to work the TV, but that’s all.

IC 55

Among the ICs interviewed, the idea of a social network for ICs raised little interest:

I think it’s good… For the moment, I don’t need it, because...Well, I have a good rapport with the home help; it’s going well.

IC 23

I’m not…I’ve never been a person like that, not very communicative and that...So, everyone has to say what they do, what they’ve got...It’s not...No, not for me. But I’m not saying that it might not be a help to a lot of people.

IC 29

Oh! It’s not bad. It could...for someone who is alone, be a help, yes.

IC 40

Brain training technology was selected by only one IC, but the quoted text below illustrates the difficulties in using this technology:

She can’t do it alone either. Well, memory exercises would be great, but maybe someone would have to help because of her not being able to use a tablet.

IC 42

To the ICs, activity sensors were not a promising technology to strengthen home support:

Ah! Well, for people who live alone, that’s very good.

IC 23

Oh, yeah, sometimes he hasn’t closed [the fridge door] properly. It’s happened sometimes, when it didn’t shut properly.

IC 29

[…] she’ll say to me: “You don’t think I’m crazy yet, do you?” All this scares me.

IC 38

Yes. Yes, well that could be, err, that could be good, indeed. Yeah because in half an hour anything can happen, eh?

IC 42

[…] I wouldn’t even trust the sensor [...].

IC 44

[…] it’s true that these are technologies that are great for those who are not safe, but who are in their right mind, and everything, who don’t have all their heads and bodies; and who may be putting themselves in danger, uh, about that.

IC 55

##### Professional Caregivers

Among PCs, perceptions about the acceptability of technology differed according to the constraints that technologies impose on the CDOAs. The more technologies focus on monitoring a CDOA’s behavior, the more ethical questions they raise. On the contrary, the more they increase a CDOA’s autonomy and safety and decrease the burden on ICs, the more the PCs promoted them. Opinions on the acceptability of some of the technologies proposed during the PEIs were as follows. The light path technology was broadly well-received by the PCs, and the following statement illustrates their favorable opinion of this technology:

A technology that should be introduced and available for all older people.

FG 1

Technologies that increase safety, such as the fall detector, were mostly preferred by the PCs:

[…] if it’s good, it can be very useful.

FG 2

The PCs showed little interest in the robot vacuum cleaner and the service robot:

[…] There’s a risk of falling over the vacuum cleaner when it’s running...

FG 4

[…] It’s interesting if it assists caregivers [gives them time to do another activity while the robot is working].

FG 1

There's a risk of replacing humans […].

FG 3

Several PCs had questions regarding the GPS bracelet technology:

[…] it’s not relevant if its only purpose is to calm the family […] .

FG 3

[…] there are ethical problems if it’s used as a means to monitor the person […].

FG 3

The touch tablet and brain training technologies were rarely chosen by the PCs. Their reluctance was expressed as follows:

You have to accompany the arrival of the tablet in the person’s environment and ensure that the person is interested in using it….

FG 4

It might be interesting to couple a tablet to the remote alarm [to enable visualizing the person’s condition if they can’t or can no longer express themselves because of cognitive disorders]...

FG 4

[…] difficult to offer if the person does not know how to use a tablet…

FG 4

...can be a prevention tool…

FG 3

Although some PCs selected the social network and activity sensor technologies, there were mixed opinions:

[Social networking] technology is not necessarily appropriate if the caregiver does not understand those technologies; it’s rather aimed at young caregivers [...].

FG 2

[…] it can make caregivers feel supported [...].

FG 2

Concerning activity sensors, this response was obtained:

[...] Pose an ethical dilemma [...]; [...] culture can be an obstacle to the use of this technology; [...] interesting technology, and install the sensors only as needed [to see if the fridge is opening in case of a heatwave; to see that people are getting out of bed]. It can be a complementary tool in food monitoring if this problem has been previously identified [...]

FG 2

When choosing the most useful technology overall, the PCs differed according to their profession. Physicians, nurses, and occupational therapists chose the fall detector, whereas nursing assistants, social workers, and direct community health providers chose social network. Table 4 presents a summary of the main topics covered.

Multimedia Appendix 3 provides detailed data on the technologies preferred by CDOAs, ICs, and PCs. Multimedia Appendix 4 presents a detailed overview of the perceived barriers to technology use among CDOAs, as well as the photographs selected during their PEIs. Multimedia Appendix 5 provides a detailed overview of CDOAs’ perceptions of technology and its facilitators.

**Table 4:** Themes and subthemes highlighted by the thematic content analysis.

Themes	Subthemes		
	CDOAs^a^	ICs^b^	PCs^c^
Usefulness and meaning of technology to support the needs of CDOAs	Useful for others in my peer group Useful later in my life Lack of meaning Technology often misunderstood	Useful depending on the CDOA’s state of health The CDOA’s openness to using technologies Technology costs	Useful to sustain the ADL^d^ Risk of creating distance in relationships with patients Fear of being replaced by technology
Strategies to increase ease of technology use among direct users and supporting stakeholders	Encouragement from children/grandchildren Financial support Increase feeling of safety	Easing the daily strain for ICs and PCs Lightening the burden of helping Fear of mistakes	Appropriate technology implementation strategy Involving relatives Increasing the autonomy of CDOAs
Acceptability of using technology to remain at home among direct and indirect users	Influenced by having a self-developed strategy to achieve purposes Difficulties using devices The cost of devices Increasing the ability to remain at home	Safety first Ease of use Staying autonomous The cost of devices	Constraints that technologies impose Ethical questions if the focus is on monitoring behavior Increasing the CDOA’s autonomy and safety Lessing the burden on ICs

^a^CDOA: community-dwelling older adults.

^b^IC: informal caregiver.

^c^PC: professional caregiver.

^d^ADL: activities of daily living.

## Discussion

### Principal Findings and Comparison With Previous Work

To the best of our knowledge, this is the first research in the field to have combined personal interviews, FGs, and PEIs in the same study. Our results show that technologies are not yet well integrated into the daily care of frail or cognitively impaired CDOAs. Although technology can certainly offer solutions to some of the specific impairments typical among CDOAs, ICs and PCs still have little knowledge about their existence and how they should use them in everyday practice. The heterogeneity of participants’ profiles made it difficult to compare this study with others involving more homogeneous samples [38,39].

Our results indicate that most participants had both positive and negative attitudes toward technology. Positive attitudes were most often related to technologies that ensured continued mobility at a lower cost and with advanced functions (a GPS bracelet). Negative attitudes of CDOAs were often associated with the risks of becoming technology–dependent and the risk of fewer or poorer interactions with ICs and PCs, the realization that they were no longer able to stay at home, potential social problems (disturbing ICs and PCs with alarms), and the financial costs of technology use. Furthermore, the findings suggest that ICs had more positive attitudes toward assisting technology and an increased acceptance of technology than the CDOAs themselves. This could be explained by the fact that the average participating IC was 14 years younger than their CDOA and thus had more contact with technology in their daily life [40]. Nevertheless, some ICs evoked their feelings of being overwhelmed by technology. An analysis of their sociodemographic profiles shows that these ICs were generally older relatives, which is in line with the hypothesis that younger ICs are more open to using technologies. Another possible explanation is related to the feeling of being burdened, which urges ICs to seek technological support to alleviate their problems [41].

Technology use was often conditioned by the multiple expected outcomes CDOAs had with regard to remaining at home, risk control by ICs and PCs, and trying to avoid hospitalization or institutionalization. In contrast, the nonuse of technology was linked to the participants’ use of their personal and individual capacities, their health, their physical and intellectual functional capacities, and the environmental barriers they encountered (eg, risk of falling). To encourage the participants to adopt technology, our findings suggest that the potential barriers to this should first be removed at the individual level (each situation is unique, including costs), then at the technological level (technological flexibility adaptable to the situation), and finally at the environmental level (life context). These results were consistent with the research conducted by Chen and Chan [30].

Results from the PC FGs revealed that they had mixed attitudes toward technology, although most were interested in specific technologies for everyday use. They highlighted that each care situation was unique, uncertain, and often complex. They were particularly interested in the development of new technologies to help with the ADL. Finally, barriers to the use of technology by PCs themselves included lack of interest, a need for training, the architectural inadequacies of house design, cost issues, and their fears of being replaced by that technology and of the risks of dehumanizing care relationships with CDOAs. Our results highlight the need to pay more attention to adapting technology to users’ personal preferences, while focusing on technologies that provide solutions to individual problems. However, to date, there is little evidence to evaluate the usefulness or effectiveness of certain technologies in home care and notably to avoid hospitalization [42]. Causal intervention research expects a coconstruction between users, inventors, researchers, and manufacturers. This would make it possible to develop technologies that meet users’ needs (eg, a device that detects falls and sends an alert) and to fulfill the commercial requirements for making those technologies accessible to large numbers of CDOAs [43]. Our findings suggest that most devices will not require significant technological breakthroughs but rather careful adaptations to the specificities of end users. Many technologies will still require an assessment of user needs and their real potential for use. Technology development must consider the somatic and psychopathological states of health of CDOAs, as mentioned in Cohen-Mansfield's work [33]. Our findings suggest that it is important to go beyond the myth that technology will replace ICs and PCs. Indeed, integrating technologies into the home care activities of PCs should be promoted, as this would give them the time to do other things [44,45]. Technologies for care practices are promoted as facilitating safety and independent living as well as avoiding or delaying institutionalization. However, there is still a gap between these goals and the complex realities in which technologies are used [46].

Technologies in home health care settings must also be adapted to the needs and concerns of PCs [44]. In line with previous research, complex surveillance technologies (eg, cameras and motion detectors) were perceived to be intrusive and as posing a high risk to privacy; they were widely rejected by home health care stakeholders [47]. PCs raised concerns that the data collected might be stored for a long time and be accessible and misused by other actors (eg, insurance companies) [13]. The complex context surrounding care for frail CDOAs is unique, uncertain, and constantly changing [48]. This study showed that ICs were often involved in the daily care of a loved one, but that they rarely used technologies. Slowly introducing effective technologies into the everyday care they give would be a way to make their support more effective, either for monitoring care or in maintaining their relationship with the CDOA. This would help to maintain the health of both CDOAs and their ICs, who often become overwhelmed by their care responsibilities, leading to stress and physical and mental exhaustion.

The development of technologies to strengthen home support and prevent the loss of autonomy is a demographic, human, social, and economic imperative; it must be accompanied by the development of multidisciplinary skills [49]. Academic institutions should supervise this development, particularly by proposing critical reflection. It should also be noted that the realities of life will force both researchers in innovative technologies and prescribers to accept that the effectiveness of a technological tool should be based primarily on pragmatic approaches that lack the scientific rigor of causality [50].

### Strengths and Limitations

The significant sample of CDOAs, ICs, and PCs involved in this study suggests that our results may be transferable to other regions of Western Europe. The PEIs made it possible to clarify perceptions and specify acceptable technologies. Another strength of this study was its use of a novel combination of methodological approaches, via one-to-one interviews, FGs, and PEIs. This approach illustrated which technologies might be acceptable and useful to CDOAs, ICs, and PCs in the context of home care support. Our research population’s heterogeneity did not permit a thorough comparison with other studies in the literature as they involved more homogeneous samples [18-20]. In addition, to the best of our knowledge, this is the first paper in the field to combine one-to-one interviews, FGs, and PEIs, which enriches the value of our results but complicates their comparison with previous studies. One limitation of this study concerns the AGGIR tool’s subjectivity when used as an overall clinical classification system of impairment among CDOAs. In contrast, other tools are more oriented toward disease and physiopathological classification (Resident Assessment Instrument-Home care). Finally, this study had some methodological limitations concerning the choices of the images shown in the PEIs. These could not always best present the different gerontechnologies to the heterogeneous research participants (CDOAs, ICs, and PCs).

### Conclusions

Despite the omnipresence of technology in modern society, research into perceptions about it and its use to improve the daily lives of frail and cognitively impaired CDOAs is poor compared with research into its impact on younger populations. Despite some evidence that technologies promote independence among CDOAs, they are often underused in daily home health care. With this in mind, this study explored the perceptions about technology’s place in home health care among CDOAs, ICs, and PCs. Our findings showed that although many technologies were available to support independent living for CDOAs, they were significantly underused. Our results also affirmed that efforts should be made to adapt technologies to the needs of CDOAs, their ICs, and PCs. It seems important to continue searching for empirical evidence of the relevance and effectiveness of new technologies. This will help to specify the most important areas requiring intervention and indications for technology use among different profiles of CDOAs, their ICs, and PCs. Technology will also help to optimize the management of CDOAs’ health problems and slow their loss of autonomy, both of which will strengthen home health care. Progress toward this goal will only be achieved through close cooperation between technical experts, home health care experts, and the end users—CDOAs, ICs, and PCs—who need the appropriate technological tools to meet their needs and expectations. Finally, as the mean age of the population is rising, the proportion of older adults with an interest in technology and with technological skills will increase. The findings in this study will enable future CDOAs to clearly express the advantages and limitations of the technologies in their lives. The current situation is therefore very fluid, and research will have to adjust to this dynamic process. Although there is no doubt that technologies will play an increasingly important role in health care services for CDOAs and in the work of their ICs and PCs, it is more difficult to predict which types of interventions may develop. Establishing convincing results based on robust scientific evidence will be difficult, but clinical research will play a key role.

## Appendix

Multimedia Appendix 1 Classification of community-dwelling older adults degree of dependence: the Autonomy Scale of Gerontology and Iso-Resource Groups model.

Multimedia Appendix 2 Interview guides.

Multimedia Appendix 3 Preferred technology of informal caregivers and professional caregivers.

Multimedia Appendix 4 Technology selected by community-dwelling older adults (n=68).

Multimedia Appendix 5 Perceptions of technology and facilitators of technology use among community-dwelling older adults (N=68).

## Abbreviations

ADL: activities of daily living
AGGIR: Autonomy Scale of Gerontology and Iso-Resource Groups
CDOA: community-dwelling older adult
FG: focus group
GIR: Groupes Iso-ressources
IC: informal caregiver
PC: professional caregiver
PEI: photo-elicitation interview
