# Supplementary material for: Perceptions About Technologies That Help Community-Dwelling Older Adults Remain at Home: Qualitative Study
Source: J Med Internet Res. 2020 Jun 4;22(6):e17930. doi: 10.2196/17930 (PMC7303826; doi:10.2196/17930)
Supplement: Multimedia Appendix 1 [file jmir_v22i6e17930_app1.docx]

**Supplementary file 1**

**Classification of CDOAs’ degree of dependence: the AGGIR model**

The Gerontological Autonomy and Iso-Resource Groups (AGGIR) model evaluates the activities that a CDOA does or does not carry out. This makes it possible to define “iso-resource groups” bringing together individuals with a similar level of need for assistance in performing the essential activities of daily living (ADL). The model includes ten physical and mental activity variables (discriminating) and seven domestic and social activity variables (illustrative). The physical and mental variables are grooming, dressing, nutrition, urinary and fecal elimination, transfers, indoor and outdoor movement, alertness, coherence, and orientation. The domestic and social activity variables are management, cooking, house cleaning, transport, shopping, treatment monitoring, and leisure activities. Some variables, such as grooming, dressing, nutrition, going to the toilet, coherence, and orientation are coded using several sub-variables (e.g., grooming the upper and lower body requires significantly different mental, motor, and functional abilities). The specific rules to be applied are indicated for each variable. There are six iso-resource groups (GIR), calculated using a complex algorithm which imposes computing:

- GIR 1 includes CDOAs confined to their bed or to a chair, who no longer have any mental, physical, locomotor, and social activity, and for whom the continuous presence of healthcare professionals is indispensable. They are no longer able to live in their own homes and are in long-term care facilities.
- GIR 2 is essentially composed of two subgroups. On the one hand are CDOAs who are confined to their bed or to a chair but who retain mental function. They are not totally impaired but require assistance for most ADL, permanent supervision, and repetitive assistance actions, by day and night. On the other hand are CDOAs whose mental functions are impaired, but who have retained their locomotor capacities and can carry out certain physical activities. Their conserved locomotor capacities require permanent supervision, interventions related to behavioral disorders, and assistance with physical activities.
- GIR 3 is mainly made up of CDOAs who have maintained satisfactory mental function and partial locomotor function. However, they require assistance several times a day for physical activities. Most of them are unable to ensure their hygiene after going to the toilet.
- GIR 4 essentially includes two subgroups. On the one hand are CDOAs who cannot transfer alone, but who, once up, can move around inside their dwelling, and who must be helped or stimulated for grooming and dressing; most can eat unassisted. On the other hand are CDOAs who have no locomotor problems, but who need help with physical activities, including meals. All the CDOAs of the two subgroups can eliminate their urinary and fecal waste, although partial and occasional help may be required (on waking up, at mealtimes, at bedtime, and occasionally at their request).
- GIR 5 is made up of CDOAs who can transfer alone, move about within their accommodation, feed and dress alone. They may require occasional assistance with ADL.
- GIR 6 brings together all the independent CDOAs who can act with discrimination in their everyday life.

Based on this framework, the PCs from the five community healthcare centers involved in the project categorized the CDOAs meeting the study inclusion criteria into three ADL groups:

- - Independent CDOAs only receiving the following home-help services: meals, cleaning, and shopping (GIR 5 and 6);
  - CDOAs with mostly physical impairments (GIR 2 to 4);
  - CDOAs with mostly cognitive impairments (GIR 2 to 4).
