# Supplementary material for: Perceptions About Technologies That Help Community-Dwelling Older Adults Remain at Home: Qualitative Study
Source: J Med Internet Res. 2020 Jun 4;22(6):e17930. doi: 10.2196/17930 (PMC7303826; doi:10.2196/17930)
Supplement: Multimedia Appendix 2 [file jmir_v22i6e17930_app2.docx]

## Supplementary file 2: Interview guides

### **Community-dwelling older adults (CDOAs)**

Opening question:

- Why do you need help daily?
- Follow-up questions (need for help):
  - Explain to me how your usual day/week goes.
  - What would help you more?

Questions targeting technology:

- Sometimes technologies are used to make life easier. What do you think?
- Follow-up questions (technology):
  - Do you think that any technologies could help you solve the problems we have just discussed?
  - What would make you want to use them?
  - On the contrary, what would hold you back?
  - Do you already use technologies or technological objects (purpose, frequency)?
  - How did you start using this technology? (the one mentioned by the interviewee during the interview).

Use of the catalogue:

- The catalogue must be used with care. The interviewee should not feel that he or she is being tested or advertised to. All photos must be presented one after the other with their respective information followed by the questions below.
- This photo (p. X, then p. Y, then p. Z, etc.) represents (respective information). What do you think about this technology? What could you use it for?

Conclusion:

- Spread out all the photos on a table
- Out of all these photos, could you choose the one that would be the most useful in your daily life?

### **Informal caregiver’s guide (ICs)**

Question targeting "help provided":

- In which daily situations do you help xxx (spouse, parent, ...)?
- Follow-up questions ("help provided"):
  - In practice, what does that consist of (type of assistance, frequency, duration)?
  - How have you dealt with these situations so far?
  - Can you give me an example of a situation where you helped xxx?
  - In these cases, what makes your life easier?
  - Do you have any personal strategies?
  - Do you receive any third-party assistance (professional or other)?
  - Do you have any other additional resources, financial help, fitting out, moving...)
  - What would help you more?

Questions targeting technology:

- Sometimes technologies are used to make life easier. What do you think about the help you give to XX?
- Follow-up questions (technology):
  - Do you think that there are any technologies that could help you solve the problems of the person you help daily?
  - How could they help you in your role as a caregiver?
  - What would make you want to use them?
  - On the contrary, what would hold you back?

Use of the catalogue:

- The catalogue must be used with care. The interviewee should not feel that he or she is being tested or advertised to. All photos must be presented one after the other with their respective information followed by the questions below.
- This photo (p. X, then p. Y, then p. Z, etc.) represents (respective information). What do you think about this technology? What could you use it for?

Conclusion:

- Spread out all the photos on a table.
- Out of all these photos, could you choose the one that would be the most useful in your daily life?

### **The PEI guide**

The PEI guide (Figure 1) was composed of ten photographs of technologies, selected by the research team and based on a classification of gerontechnology (7) and their empirical expertise.

| Photo 1: Light path  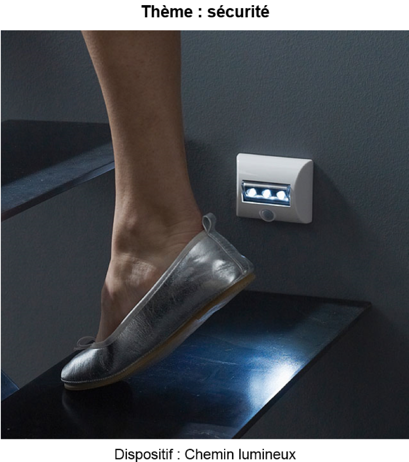 | Photo 2: Fall detector  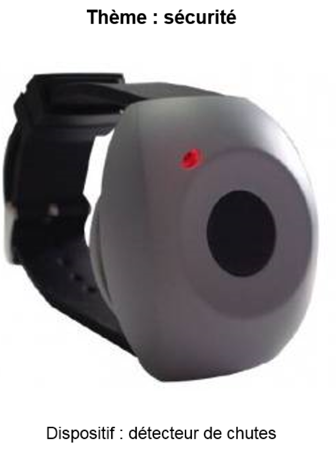 | Photo 3: Electronic pillbox  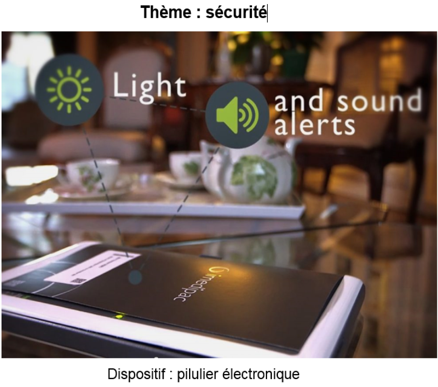 |
| --- | --- | --- |
| Photo 4: Robot vacuum cleaner  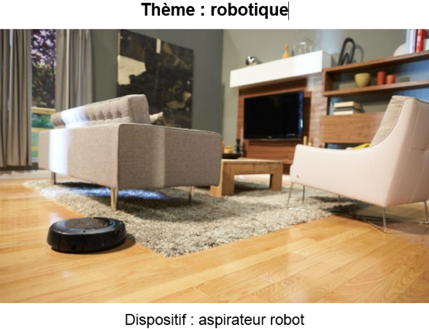 | Photo 5: Service robot  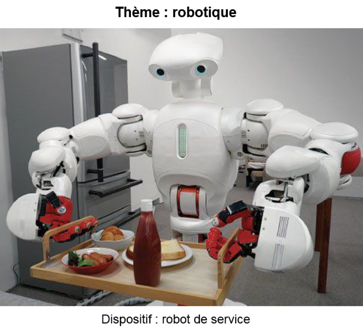 | Photo 6: GPS bracelet  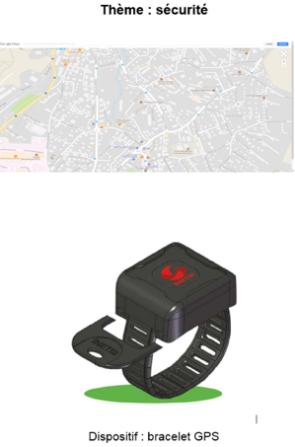 |
| Photo 7: Touchscreen tablet  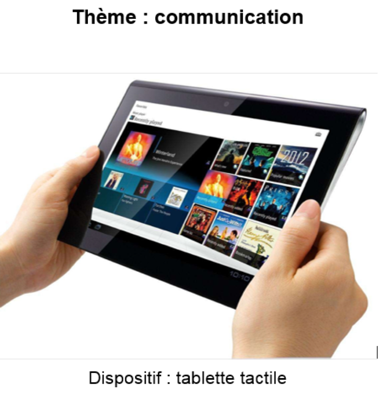 | Photo 8: Social network  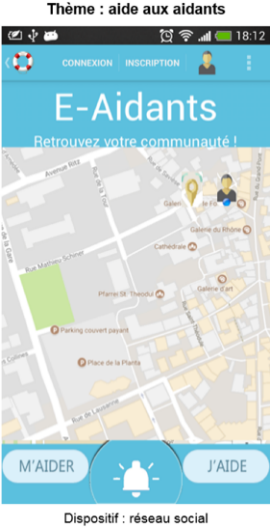 | Photo 9: Brain training  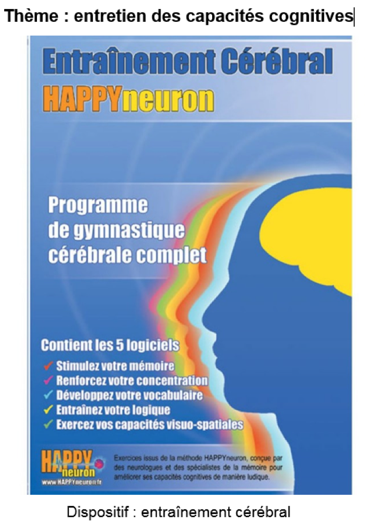 |
| Photo 10: Activity sensor  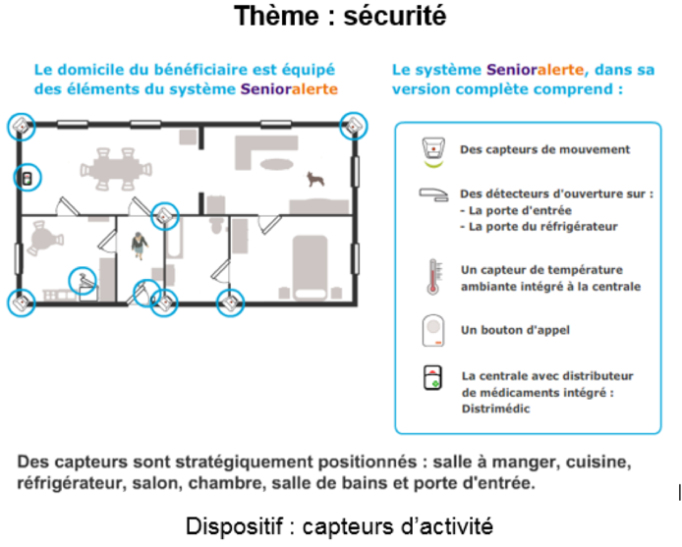 | | |

Figure 1: Technologies included in the PEI approach.
