# Supplementary material for: Perceptions About Technologies That Help Community-Dwelling Older Adults Remain at Home: Qualitative Study
Source: J Med Internet Res. 2020 Jun 4;22(6):e17930. doi: 10.2196/17930 (PMC7303826; doi:10.2196/17930)
Supplement: Multimedia Appendix 3 [file jmir_v22i6e17930_app3.docx]

## Supplementary file 3

### Preferred Technology of ICs

ICs selected technologies related to the safety of their loved ones. This might be explained by the fact that of the 21 ICs interviewed, 12 were relatives of a CDOA with a mainly cognitive impairment. Thus, the technologies selected by all ICs join those of this group of mainly impaired cognitive older adults. ICs’ opinions on the different technologies presented during the PEIs were as follows. Several ICs selected the light path, saying: “Yes, I think that’s really good. Well, because my mum gets up at night and she can't find the light” (IC 42); “That's good, because I see that he gets lost when he goes to the toilet” (IC 44); “Maybe it could... yes, a light path, like that… to go to the bathroom sometimes, that's right behind the bedroom.” The fall detector was selected by about one third of ICs: “Yes, that [the fall detector] could be the most useful one there. In the future there may be others” (IC 43); “Maybe rather the fall detector, I'd say, then. A fall detector for when you pass out when you fall down” (IC 55). ICs expressed some reticence about electronic pill dispensers. “Ah, well, that's (laughs) what they’re made for, it might be useful because I want him to have a pill box; the nurses come to put the pills in. But if he doesn't think about it—or if I don't think about it, he doesn't think about it—then it's useless” (IC 29) ; “Yeah, it's not a bad thing, but I think it's pretty expensive for the use we make of it. When we're at home, well, they’re taken at mealtimes, so I'm here (laughs)” (IC 40); “In my mother’s case, she’d be unable to take her medication alone.” ICs showed divergent opinions about the robot vacuum cleaner: “For me, these robots are good. You shouldn't leave anything on the ground (laughs). No, you shouldn't. It can't get into the corners: it's round (laughs)” (IC 29); “Well, it depends on how much it costs, eh?” (IC 38); “It’s not essential, in my opinion” (IC 40); “I don’t think so, no. It might be more dangerous than useful” (IC 42). ICs’ opinions on the service robot diverged too: "Listen, yes, it's good, but in the end a human-being is still better, that’s my feeling, eh?” (IC 23); “I find it rather stressful” (IC 29); “Well, it's already... (laughs) I just see something cumbersome” (IC 38). Opinions on the usefulness of a GPS bracelet were summed up by IC 38: “Me, I’m all for that. If she ever decided to go off somewhere, at least she’d know... we’d know where she was, eh?” (IC 38). Although only a fraction of the ICs selected the touchscreen tablet as an interesting technology, there were divergent opinions: “No, so mum, my mother... she's not interested”(IC 23); “It might make things easier for him, but since he's not very passionate about it, he quickly lost patience” (IC 29); “Err, no (laughs). That, I think, is just not possible. Because even a mobile phone would be complicated to use, so... There you are. She still manages to get the TV to work, but that's all” (IC 55). The social network for ICs seemed to raise little interest among the ICs interviewed: “I think it's good, but for the moment, I don't need it, because... well, I have a good relationship with the home help, it's going well” (IC 23); “I'm not, I've never been a very communicative person like that... And then everyone has to say what they do. No, it's not for me. After all, though, it might help a lot of people out” (IC 29); “Oh! It's not bad. It could... for someone who’s alone, to help, yes” (IC 40). Brain training technology was only selected by one IC, and IC 42 pointed out the difficulties in using this technology: “She can't do it alone, either. Well, memory exercises would be great, but maybe someone could make her do it as she’s not capable of using a tablet” (IC 42). ICs did not select activity sensors as a promising technology to strengthen home support: “Ah! Well, for people who live alone, it's very good” (IC 23); “Oh, yeah. Sometimes he doesn't close the [fridge] door properly (IC 29); “She’ll tell me, ‘You don't think I'm crazy yet, do you?’ And all that scares me” (IC 38); "Yes, yes, well that could be good, indeed, because anything can happen in half an hour, eh?” (IC 42); “I wouldn't even trust the sensor” (IC 44); “It's true that these are technologies that are great for those who are not safe, who’ve not got all their mental faculties and everything, or who are a bit mentally and physically impaired and who might put themselves in danger” (IC 55).

### Preferred Technologies among PCs

PCs’ opinions differed according to the constraints that technologies imposed on CDOAs. The more technologies focus on monitoring CDOA behavior, the more they bring up ethical questions. PCs broadly selected the light path, with many favorable opinions on this technology: “A technology that should be introduced into all older people’s homes” (FG 1). The fall detector was the technology most selected by the PCs: “If a solution is ready, it could be very useful” (FG 2). There were unfavorable opinions on the electronic pill dispenser, however: “It’s complicated to use for caregivers”, “Care is a little bit dehumanized”, “It’s interesting for highly fragmented treatments (like Parkinson's) and for reminding patients with cognitive disorders to take their medication” (FG 3). PCs expressed little interest in the robot vacuum cleaner and the service robot: “There’s a risk of falling when the vacuum cleaner is running” (FG 4); “It’s interesting if it’s assisting caregivers (giving them time to do another activity while the robot’s working)” (FG 1); “There’s a risk of replacing the human touch” (FG 3). Several PCs raised questions about the GPS bracelet technology: “It’s not appropriate if its only purpose is to reassure the family” (FG 3); “There’s an ethical problem if it’s used as a means to monitor the person” (FG 3).

Few PCs selected the touchscreen tablet and brain training technologies, and the others expressed their reluctance to do so: “The tablet’s arrival in the person's environment has to be accompanied and that person has to be interested in using it” (FG 4). “It would be interesting to link this to the remote alarm (enabling us to visualize the person's condition after a malaise if they can't or can no longer express themselves because of cognitive disorders” (FG 4); “It’s hard to propose a tablet if the person doesn’t know how to use one” (FG 4); “It could be a preventive tool” (FG 3).

Although some PCs selected the social network and activity sensor technologies, opinions varied among the participants. For the social networking website: “The technology might not necessarily be appropriate if the caregiver doesn’t master technologies which have been developed for young caregivers” (FG 2); “Can make caregivers feel supported” (FG 2). Activity sensors: “They pose an ethical dilemma”, “Culture can be an obstacle to technology use”, “It’s an interesting technology and sensors can be installed as needed (Is the fridge opening during heat waves? Is the person getting out of bed? Is the person sleeping?), as a complementary tool in food monitoring if this is a previously identified problem” (FG 2). The technology selected by PCs differed according to their professional role. Physicians, nurses, and occupational therapists chose the fall detector. Nursing assistants, social workers, and providers of community healthcare chose the social network.
