# Supplementary material for: Perceptions About Technologies That Help Community-Dwelling Older Adults Remain at Home: Qualitative Study
Source: J Med Internet Res. 2020 Jun 4;22(6):e17930. doi: 10.2196/17930 (PMC7303826; doi:10.2196/17930)
Supplement: Multimedia Appendix 4 [file jmir_v22i6e17930_app4.docx]

## Supplementary File 4

Supplementary Table. Technology selected by CDOAs (n = 68)

|  | *Physically impaired* | *Cognitively impaired* | *Functionally independent* | **Total** |
| --- | --- | --- | --- | --- |
|  | Number | Number | Number |  |
| Light path (%) | 2 | 1 | 4 | **7 (12)** |
| Fall detector (%) | 6 | 6 | 3 | **15 (25)** |
| Electronic pillbox (%) | 1 | 0 | 0 | **1 (2)** |
| Robot vacuum cleaner (%) | 2 | 2 | 3 | **7 (12)** |
| GPS bracelet (%) | 0 | 6 | 4 | **10 (17)** |
| Touchscreen tablet (%) | 0 | 1 | 2 | **3 (5)** |
| Brain training (%) | 5 | 4 | 3 | **12 (19)** |
| Activity sensor (%) | 2 | 1 | 2 | **5 (8)** |
| **Total** | **18** | **21** | **21** | **60 (100)** |

Note: The service robot and social networking website were not selected by CDOAs, and thus are not presented in this table.
