# Supplementary material for: Perceptions About Technologies That Help Community-Dwelling Older Adults Remain at Home: Qualitative Study
Source: J Med Internet Res. 2020 Jun 4;22(6):e17930. doi: 10.2196/17930 (PMC7303826; doi:10.2196/17930)
Supplement: Multimedia Appendix 5 [file jmir_v22i6e17930_app5.docx]

Supplementary file 5 : perceptions of technology and facilitators of technology use among CDOAs (N=68)

Table 5. Overview of the perceptions of technologies among CDOAs (n = 68)

| **Perceptions of technologies** | | |
| --- | --- | --- |
| ***Physically-impaired*** | ***Cognitively-impaired*** | ***Independent*** |
| Useful, but for other health problems (5)  Useful, but not now (4)  Misunderstood (4)  *A priori* negative (4)  *A priori* positive (3) | *A priori* positive (4)  *A priori* negative (3)  Useful, but not now (3)  Useful, but for other health problems (3)  Misunderstood (3)  Useful, but for someone else (1) | Misunderstanding (5)  *A priori* negative (4)  Useful, but for other people (3)  *A priori* positive (3)  Useful, but for other health problems (2)  Useful, but not now (1)  Useful now (1) |

Note. The bracket shows the number of times a perception was mentioned.

Table 6. Facilitators of technology use among CDOAs (N = 68)

| **Technology-use facilitators** | | |
| --- | --- | --- |
| ***Physically-impaired*** | ***Cognitively-impaired*** | ***Functionally independent*** |
| Feeling of improved safety (2)  Financial assistance (2)  Help with device-use from a family member (2)  Recommended by healthcare professionals or the home help (2)  Chosen by a family member (2)  Device was a present (2)  Lessons on how to use the device (2)  Help in requesting the device (1)  In favor of progress (1)  Had previously used a device (1)  Knowing about the device and being able to test it (1)  Device on loan (1)  Learning how to use the device (1) | Used with a family member (2)  Device usefulness (1)  Help with device-use from a family member (1)  Ease of use (1)  Chosen by a family member (1)  Device was a present (1)  Financial assistance (1)  Recommended by healthcare professionals or the home help (1)  Feeling of improved safety (1) | Feeling of improved safety (5)  Device was a present (3)  Help in using the device (3)  Ease of use (3)  Previous use by a spouse (1)  Chosen by a family member (1)  Solution to a health problem (1)  Had previously used a similar device (1)  Lessons on how to use the device (1)  Had previously used technologies (1)  Recommended by healthcare professionals or the home help (1) |
